# Endogenous hormone 2‐methoxyestradiol suppresses venous hypertension‐induced angiogenesis through up‐ and down‐regulating p53 and id‐1

**DOI:** 10.1111/jcmm.13399

**Published:** 2017-11-29

**Authors:** Xiang Zou, Li Zhang, Jie Yuan, Chunjie Yang, Zehan Wu, Jianping Song, Wei Zhu, Ying Mao, Liang Chen

**Affiliations:** ^1^ Department of Neurosurgery Huashan Hospital Fudan University Shanghai China; ^2^ Shanghai Institute of Cardiovascular Diseases Zhongshan Hospital Fudan University Shanghai China; ^3^ State Key Laboratory of Medical Neurobiology School of Basic Medical Sciences Institutes of Brain Science Fudan University Shanghai China

**Keywords:** angiogenesis, arteriovenous malformation, 2‐Methoxyestradiol, HIF‐1α, ID‐1, p53

## Abstract

Brain arteriovenous malformations (AVMs) which associate with angiogenesis due to local hypertension, chronic cerebral ischaemia and tissue hypoxia usually lead to haemorrhage, however, the therapeutic medicine for the disease is still lacking. 2‐methoxyestradiol (2‐ME) has been shown effective in the anti‐angiogenic treatment. This study was conducted to examine whether and how 2‐ME could improve the vascular malformations. Intracranial venous hypertension (VH) model produced in adult male Sprague‐Dawley rats and culture of human umbilical vein endothelial cells (HUVECs) at the anoxia condition were used to induce *in vivo* and *in vitro* angiogenesis, respectively. Lentiviral vectors of *ID‐1* and *p53* genes and of their *siRNA* were intracranially injected into rats and transfected into HUVECs to overexpress and down‐regulate these molecules. 2‐ME treatment not only reduced the *in vivo* progression of brain tissue angiogenesis in the intracranial VH rats and the *in vitro* increases in microvasculature formation, cellular migration and HIF‐1α expression induced by anoxia in HUVECs but also reversed the up‐regulation of ID‐1 and down‐regulation of p53 in both the *in vivo* and *in vitro* angiogenesis models. All of the anti‐angiogenesis effects of 2‐ME observed in VH rats and anoxic HUVECs were abrogated by ID‐1 overexpression and p53 knockdown. Our data collectively suggest that 2‐ME treatment inhibits hypoxia/anoxia‐induced angiogenesis dependently on ID‐1 down‐regulation and p53 up‐regulation, providing a potential alternative medical treatment for un‐ruptured AVM patients.

## Introduction

AVMs are congenital cerebrovascular nidus that often cause haemorrhage in young adults. Spontaneous bleeding, growth and recurrence indicate that brain AVMs are lesions with active angiogenesis and vascular remodelling 1, 2, 3, 4. Although brain AVMs is considered to be congenital, the underlying mechanisms of progression are still unclear. Wall shear stress has been implicated as an important biomechanical stimulus for vascular remodelling associated with cerebral AVMs 5, but it may not be the sole cause 2. Although vessels in the nidus are exposed to arterial blood therefore unlikely to be ischaemic, steal phenomenon may induce hypo‐perfusion instead in surrounding tissue. Local chronic cerebral ischaemia and tissue hypoxia will lead to the angiogenesis and vascular malformations 6, 7. For this reason, rodent intracranial VH model is widely used to study human brain AVMs and dural arteriovenous fistulas (DAVFs).

Intracranial VH is mainly caused by sinus thrombosis, contributing to various cerebrovascular diseases such as DAVFs 6. In our previous studies, we verified the hypothesis that some acquired human intracranial vascular malformations can be caused by VH through a rat intracranial VH model. We found that angiogenesis was highly active in the dura mater after modelling, which finally created a gross malformed vascular mass 8. Following works using the same model further confirmed the local hypoxia and angiogenesis status, marked by relevant factors such as vascular endothelial growth factor (VEGF), matrix metalloproteinases (MMPs) and hypoxia‐inducible factor‐alpha (HIF‐1α) 9. Besides, differentiation‐1 (ID‐1) as a transcription regulator was also found to synchronously change with HIF‐1α *in vivo* 9, similar with the findings in oncology studies 10. In addition, the level of p53 became lower in the brain tissue under chronic intracranial VH, in our pilot experiments. The susceptibility of intracranial vascular malformations has already been proved to relate to sex hormones. Patients of DAVF had lower serum estradiol levels than control patients 11. Although estradiol does not have a direct anti‐angiogenesis effect, its metabolite 2‐ME is a potent agent 12. Because of its low binding affinity to oestrogen receptors, 2‐ME does not exhibit direct oestrogenic activity compared to estradiol 13. Moreover, we recently found that the increasing level of ID‐1, HIF‐1α together with angiogenesis factors and microvessel density could be reduced by 2‐ME 9.

Although the origin of intracranial VH can sometimes be cured, vascular malformations such as AVMs and DAVFs are more thorny problems. Surgery, endovascular intervention and radiotherapy are primary treatments for these malformations, but the complexity of the vascular architecture often limits the effectiveness of those therapies 14. Moreover, high‐grade malformations are more likely to present with haemorrhage 15. Considering the previous findings in the rat intracranial VH model, we speculated that anti‐angiogenesis treatment is a reasonable choice for adjuvant therapy. The aim of this study was to assess the role of ID‐1 and p53 in regulation of HIF‐1α, and the mechanism of 2‐ME in attenuating angiogenesis in the intracranial VH rat model and anoxic HUVECs.

## Materials and methods

### Surgical and intervention protocols

Experimental protocols for animals were approved by the Huashan Hospital Institutional Board, Fudan University, Shanghai, China. Study design was in accordance with the ARRIVE guidelines. Fifty two male Sprague‐Dawley rats weighing 300–350 g, aged about 11 weeks were used in this study. All rats were bred and kept in a pathogen‐free animal facility. Rats underwent surgical procedures after intraperitoneal injection of 10% chloral hydrate at a dose of 0.2 ml/100 g and with 1% lidocaine local anaesthesia. Surgical procedures for rat VH model were described in our previous studies 8, 9.

### Animal grouping

Rats were randomly grouped into eight groups. Sham group: rats were only exposed with external jugular vein and common carotid artery. Sham/2ME group: rats after sham operation were treated by 2‐ME for 28 days. VH group: rats were received VH modelling. VH/2ME group: rats after VH modelling were treated by 2‐ME for 28 days. VH/ID‐1+ group: rats were received VH modelling and right basal ganglia injection of ID‐1 overexpression lentiviral vector. VH/ID‐1+/2ME group: rats were received VH modelling and right basal ganglia injection of ID‐1 overexpression lentiviral vector, then treated with 2‐ME for 28 days. VH/p53‐ group: rats were received VH modelling and right basal ganglia injection of shRNA of p53 lentiviral vector. VH/p53‐/2ME group: rats were received VH modelling and right basal ganglia injection of shRNA of p53 lentiviral vector, then treated with 2‐ME for 28 days. The dose of 2ME was 60 mg/kg/day, dissolved by DMSO as 300 mg/ml. All the animals underwent bypass surgery were checked for vascular patency by dissection before sampling. Rats with vascular occlusion were excluded. Intervention, vessel dissection and sampling were performed by blinded investigators. Animal modelling and grouping diagram were presented in Figure S1.

### Local lentivirus vectors injection

The peri‐cranial muscles and fascia were retracted laterally after a midline scalp incision. Next, a bone hole (1 mm diameter) was drilled 1 mm posterior and 4 mm lateral to the bregma following the coordinates of the stereotaxic atlas. Through this hole, 100 μl lentivirus vectors (10^9^ pfu) were injected over a period of 5 min. into basal ganglia at a depth of 5 mm using a microinjection syringe. The needle was held in place for an additional 60 sec. to prevent reflux.

### Cell preparation, culture, anoxia incubation and 2‐ME treatment

The primary HUVECs were isolated from fresh human umbilical veins of 32–34 weeks old and seeded on a 6‐well plate in Endothelial Cell Medium (ECM, ScienCell Research Laboratories, Santiago, CA, USA) containing 5% FBS, 100 U/ml P/S solution and 100 U/ml ECGs at 37℃ and 5% CO_2_. The 2–3 passages of HUVECs were used and cultured at 5 × 10^5^/well in this study. Before treatments, HUVECs were cultured in serum‐free ECM for 24 hrs. Anoxia culture was performed using a three gas incubator (1% O_2_). 2‐ME (Sigma‐Aldrich, St. Louis, MO, USA) diluted with DMSO was added to HUVECs at the concentration of 10 μM.

### Lentiviral vectors and cellular transfection

Construction of lentiviral vectors encoding *p53* and *ID‐1* has been previously described 10, 16. Small interfere RNA (*siRNA*) of *ID‐1* and *p53*, and their scramble sequences were synthesized by PCR based method and purchases from Genepharma, Inc. (Shanghai, China). These constructs were inserted into lentiviral vectors (Invitrogen, Carlsbad, CA, USA). Transfection of lentiviral vectors encoding *ID‐1* and *p53*, and their *siRNA* into cultured cells were performed as reported previously. Efficacy of adenoviral vector transfection was evaluated by examining the ID‐1 and p53 protein expressions using Western blotting.

### Cell migration assay

HUVECs transfected with relative lentivirus vectors were seeded at 1 × 10^5^/well in the plates with an Ibidi‐silicone insert (Ibidi, Martinsried, Germany) according to the product instruction. This insert allows for the formation of a well‐defined ‘edge’ without physically scratching or wounding the cell monolayer. The cells were cultured for 24 hrs to form a confluent monolayer prior to careful removal of the insert. After removal of the insert using sterile tweezers, cells were then rinsed twice with media, and fresh ECM with or without 2‐ME was added to the dish prior to the anoxia or normoxia incubation. After 24 hrs incubation, the cells were observed under a microscope (×25). The NIH image processing software was used to measure the cell‐covered area.

### Tube formation experiments

HUVECs transfected with relative lentivirus vectors were resuspended in 2% FBS‐ECM, implanted onto the matrigel (BD Inc., San Jose, CA, USA) in 24‐well plate in a density of 5 × 10^4^ cells/well and grown for 18 hrs under the normoxia or anoxia condition with or without 2‐ME pre‐treatment for 24 hrs. The capillary formation was observed under a microscope (×100) and quantified by calculating the total numbers of branch points in whole well.

### Immunohistochemistry staining

Rabbit polyclonal antibodies against VEGF (1:250 dilution; Abcam, Cambridge, UK, ab46154), MMP‐9 (1:100 dilution; Abcam, ab7299), ID‐1 (1:200 dilution; Abcam, ab134163), p53 (1:100 dilution; Abcam, ab131442) and HIF‐1α (1:200 dilution; Abcam, ab51608) were used for immunohistochemical staining. The secondary antibody was goat anti‐rabbit immunoglobulin (1:500 dilution; Abcam).

### Immunofluorescent staining

Rabbit polyclonal antibodies against CD31 (1:200 dilution; Abcam, ab64543) were used for immunofluorescent staining. The secondary antibody was goat anti‐rabbit immunoglobulin (1:500 dilution; Abcam). Nuclei were visualized with 4′,6‐diamidino‐2‐phenylindole (DAPI). Slides were photographed for red (Alexa Fluor 594), green (Alexa Fluor 532) and blue (Alexa Fluor 488) fluorescence with a fluorescent microscope (Olympus, Tokyo, Japan). Microvessels marked by CD‐31 (lumen diameter < 10 cells) were measured in five random 200x fields in five sections, and the microvessel density (MVD) was calculated. The assessment of the micro vessels was performed by two independent observers who were blinded to each group.

### Western blot analysis

Total proteins extracted from the tissues or cells were subjected to Western blot analysis for HIF‐1α (1:1000 dilution; Abcam, ab51608), ID‐1 (1:1000 dilution; Abcam, ab134163), p53 (1:1000 dilution; Abcam, ab131442), VEGF (1:1000 dilution; Abcam, ab46154), MMP‐9 (1:1000 dilution; Abcam, ab7299) or GAPDH (1:2000 dilution; Boster, BM3876) in standard routine with relative antibodies (Santa Cruz Biotechnology, CA, USA). Quantitative analysis was performed by LAS‐3000 imaging system (FUJIFILM Inc, Tokyo, Japan).

### Statistical analyses

All statistical significance was determined by one‐way or two‐way anova with Newman–Keuls test for post hoc analysis. A *P* value of < 0.05 was considered statistically significant. All results are presented as mean ± S.D. of the mean.

## Results

### Effects of 2‐ME on the expression of ID‐1 and P53 proteins in the basal ganglia of VH rats and HUVECs

**Figure 1:**
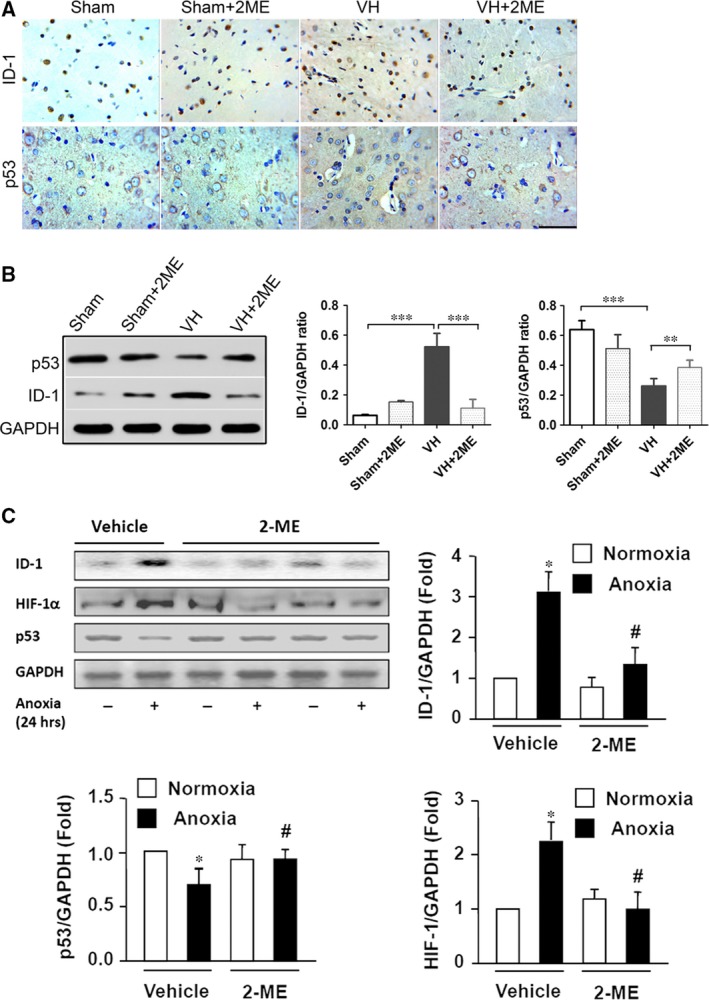
The effects of 2‐ME treatment on the expression ID‐1 and p53 proteins in the basal ganglia after intracranial VH modelling.(**A**) Immunohistochemistry staining of ID‐1 and p53 proteins in basal ganglia of the Sham and intracranial VH rats, with or without 2‐ME treatment. Scale bar: 50 μm. (**B**) Western blot analysis of ID‐1 and p53 proteins in the rats of Sham, Sham+2ME, VH and VH+2ME groups. **P <* *0.01, ***P <* *0.001; *n* = 6 per group; mean ± S.D. (**C**) Expressions of ID‐1, p53 and HIF‐1α proteins were quantified as folds of GAPDH expression in the cells subjected to normoxia with vehicle. Data are expressed as mean ± S.D. of six independent experiments (*n* = 6). *P <* *0.05 *versus* normoxia with Vehicle; ^#^ *P *<* *0.05 *versus* Anoxia with Vehicle.

As ID‐1 and p53 have been known to be the important regulators for angiogenesis, we therefore first examined the effects of 2‐ME on ID‐1 and p53 protein expressions in the basal ganglia of rat VH model. The mortality among the VH rats was 10% (4/40). Four rats died secondary to operator error or surgical failure during VH modelling. VH was successfully induced by the surgical bypass procedure in 48 rats and a sham operation was performed on the other 12 rats. The patency of the bypass was confirmed by dissection during tissue sampling. Expression levels of ID‐1 and p53 in the basal ganglia were analysed among sham and VH groups with and without 2‐ME intervention. Compared with those in the Sham group, the protein expression levels of ID‐1 were markedly higher and of p53 were obviously lower in the VH rats (Fig. 1A and B). Interestingly, 2‐ME treatment not only suppressed the increase in the expression of ID‐1 but also ameliorated the down‐regulation of p53 expression significantly in VH rats (Fig. 1A and B). In addition, the levels of ID‐1, p53 and HIF‐1α were analysed on HUVECs with or without anoxic culture and 2‐ME treatment (Fig. 1C). As a hypoxia sensitive factor, HIF‐1α level was remarkably raised in response to anoxia culture for 24 hrs as we expected in HUVECs (Fig. 1C). By 2‐ME pre‐treatment for 24 hrs, HIF‐1α level was significantly lower than vehicle treated HUVECs. For ID‐1, Western blotting illustrated the similar variation trends (Fig. 1C). Furthermore, p53 levels were presented with opposite changes in response to anoxia and 2‐ME treatment (Fig. 1C).

### The roles of ID‐1 and P53 in the inhibitory effects of 2‐ME on intracerebral angiogenesis in VH rats

**Figure 2:**
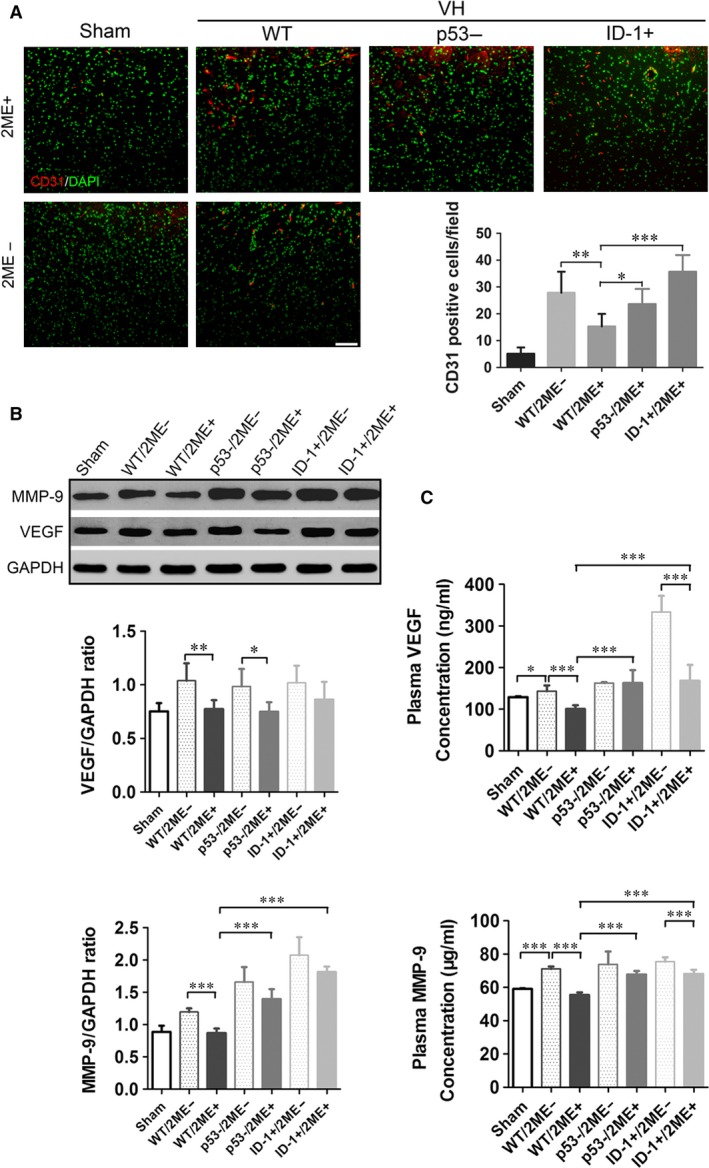
The effectiveness of 2‐ME on MVDs as well as angiogenesis factors in the basal ganglia after intracranial VH modelling. (**A**) Immunofluorescence stain of CD31 in basal ganglia of the Sham, VH, VH/ID‐1+ and VH/p53‐ group, with or without 2‐ME treatment, and MVDs analysis. Scale bar: 100 μm; red: CD31, green: DAPI. *P <* *0.05, **P <* *0.01, ***P <* *0.001; *n* = 6 per group; mean ± S.D. (**B**) Western blot analysis of VEGF and MMP‐9 proteins in the Sham, VH, VH/ID‐1+ and VH/p53‐ group, with or without 2‐ME treatment, is shown. *P <* *0.05, **P <* *0.01, ***P <* *0.001; *n* = 6 per group; mean ± S.D. (**C**) Plasma VEGF and MMP‐9 levels in the Sham, VH, VH/ID‐1+ and VH/p53‐ group, with or without 2‐ME treatment. *P <* *0.05, ***P <* *0.001; *n* = 6 per group; mean ± S.D.

We therefore asked whether the changes of ID‐1 and p53 expression play roles in 2‐ME‐exerted inhibitory effects on intracerebral angiogenesis in VH Rats. The microvessel density (MVD) in the basal ganglia of rats was measured to verify angiogenesis. In the VH group, MVDs were elevated significantly compared with Sham group, and was attenuated by 2‐ME intervention (Fig. 2A). Also, angiogenesis factors such as VEGF and MMP‐9 in the local tissue and plasma were changed synchronously with MVDs, indicating the contribution of these factors to intracerebral angiogenesis (Fig. 2B and C). Up to now, those findings were similar with our previous study, in which rats were observed during a longer time length. Interestingly, by local overexpression of ID‐1 (ID+) or knockdown of p53 (p53‐), the inhibitory effects of 2‐ME on intracerebral angiogenesis in VH rats were abolished (Fig. 2A). MVD was increased, but the inhibition of MVD by 2‐ME became weaker in VH/ID+ group. Similar phenomenon was observed in VH/p53‐ group. In addition, ID‐1 overexpression or p53 knockdown could significantly enhance the expression of MMP‐9 but not VEGF after VH modelling. However, the effect of 2‐ME on VEGF and MMP‐9 was attenuated either by ID‐1 overexpression or p53 knockdown (Fig. 2B and C). These results suggest that 2‐ME exerts inhibitory effects on intracerebral angiogenesis in VH Rats dependently on ID‐1 down‐regulation and p53 up‐regulation.

### Involvement of ID‐1 and P53 in 2‐ME‐induced inhibition of HIF‐1α expression in VH rats

**Figure 3:**
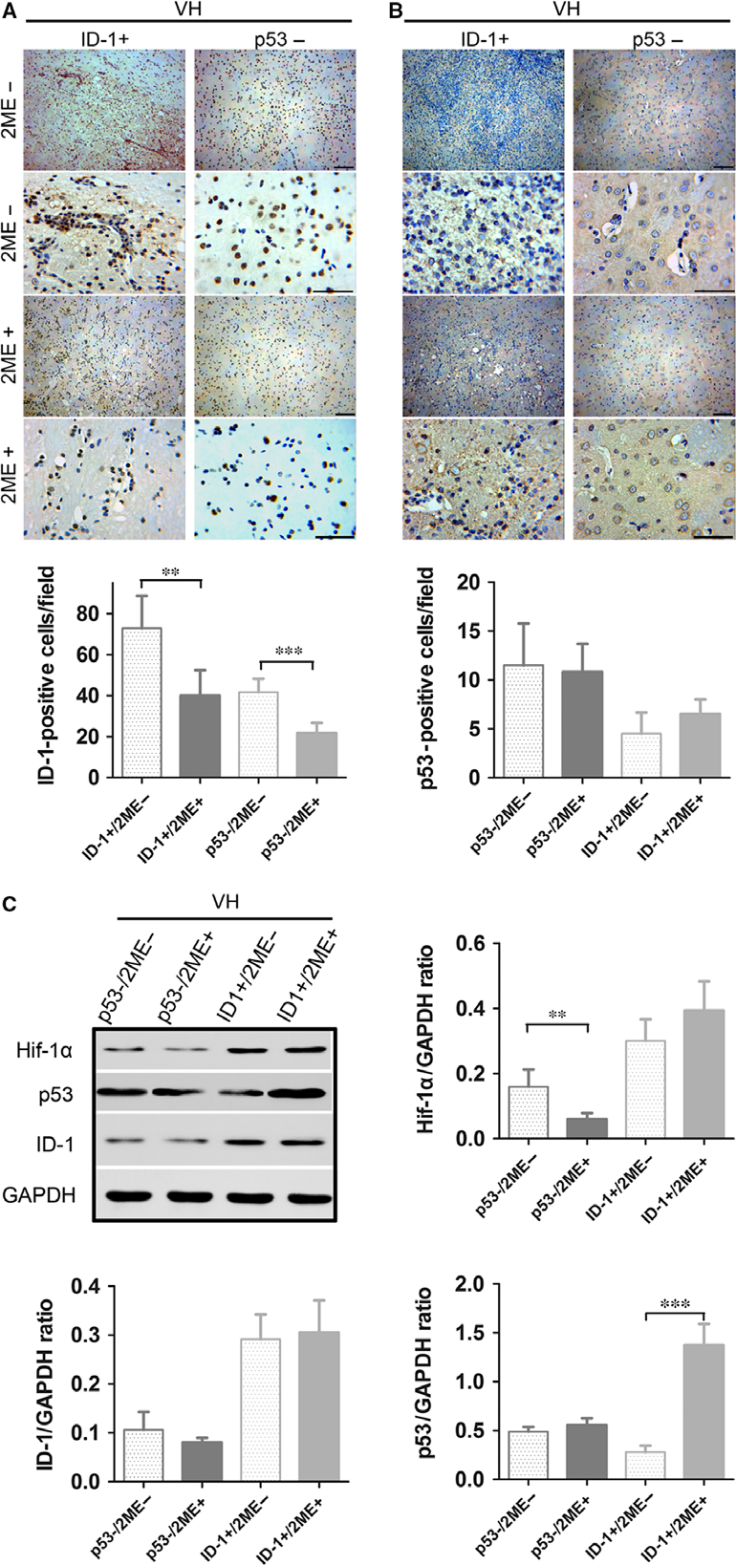
The effects of ID‐1 and p53 up‐ and down‐regulation on 2‐ME‐induced changes in ID‐1 and p53 protein expression in the basal ganglia after intracranial VH modelling. (**A, B**) Immunohistochemistry staining of ID‐1 and p53 proteins in basal ganglia and count of ID‐1 and p53‐positive cells in basal ganglia in the VH/ID‐1+ and VH/p53‐ group, with or without 2‐ME treatment. Scale bar: 50 μm. **P <* *0.01, ***P <* *0.001; *n* = 6 per group; mean ± S.D. (**C**) Western blot analysis of HIF‐1α, ID‐1and p53 protein in the VH/ID‐1+ and VH/p53‐ group, with or without 2‐ME treatment. Representative bands are shown. **P <* *0.01, ***P <* *0.001; *n* = 6 per group; mean ± S.D.

Given the above, we hypothesized that ID‐1 elevation and p53 depression after VH modelling might regulate angiogenesis, by enhancing the expression of angiogenesis factors. Then, HIF‐1α protein expression level was detected in this VH model. By immunohistochemistry staining, the number of ID‐1 or p53‐positive cells in the basal ganglia was examined among the VH rats with or without local ID‐1 overexpression and p53 knockdown (Fig. 3A and B). Also, both local ID‐1 overexpression and p53 knockdown were confirmed in the brain tissue by Western blotting (Fig. 3C). Thereafter, HIF‐1α expression was examined by Western blotting. 2‐ME treatment suppressed VH modelling‐induced elevation of HIF‐1 expression, and either overexpression of ID‐1 or knockdown of p53 significantly weakened the effect of 2‐ME on HIF‐1α suppression (Fig. 3C).

### ID‐1 and P53 regulated 2‐ME‐induced inhibitory effects of tube formation and migration in anoxic HUVECs

**Figure 4:**
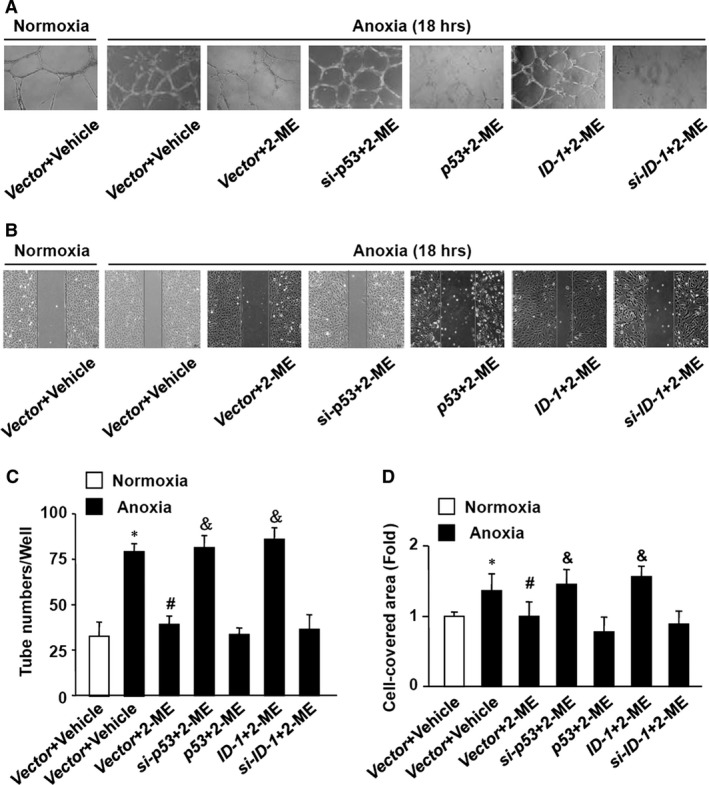
The effects of 2‐ME on microvascular formation and migration of HUVECs induced by anoxia. The HUVECs were transfected with ID‐1, p53 genes, their siRNA or empty vectors (Vector) for 24 hrs. The cells were then added with 2‐ME or vehicle and subjected to tube formation (**A**) and cellular migration (**B**) experiments under the condition of normoxia or anoxia. Representative photographs of vasculatures (**A**) and cellular migration (**B**) are shown. *si‐p53* and *‐ID‐1*, siRNA of p53 and ID‐1, respectively. (**C**) Total branch points were counted in whole dish. (**D**) Cell‐covered areas were measured in whole dish and expressed as folds of the cells transfected with empty vector and subjected to vehicle treatment under the normoxia condition. Data are expressed as mean ± S.D. of six independent experiments (*n* = 6). *P <* *0.05 *versus* Vector+Vehicle with normoxia; ^#^ *P *<* *0.05 *versus* Vector+Vehicle with Anoxia; ^&^ *P *<* *0.05 *versus* Vector+2‐ME with Anoxia.

Although the results given partially showed the role of ID‐1 and p53 in 2‐ME‐exerted inhibitory effects on angiogenesis in brain of VH rats, the complex cell compositions in the basal ganglia may confuse the behaviour of endothelial cells we mainly concerned about. We therefore employed anoxic culture of HUVECs to elucidate the mechanism. Tube formation assay and wound healing test were first performed on HUVECs (Fig. 4A and B). After anoxic culture for 18 hrs, microvasculature was significantly increased compared with normoxic HUVECs, which was obviously suppressed by 2‐ME meanwhile. However, the inhibitory effect of 2‐ME on anoxia‐induced tube formation was weakened either in shRNA‐p53‐ or ID‐1‐transfected HUVECs. In addition, there was no such phenomenon in p53 or shRNA‐ID‐1/HUVECs (Fig. 4C). Similar findings were apparent in wound healing test (Fig. 4D). An attenuated effect of 2‐ME was observed both in shRNA‐p53 and ID‐1/HUVECs, while p53 overexpression or ID‐1 knockdown is invalided. These results suggest that the inhibitory effects of 2‐ME on anoxia‐induced angiogenesis responses in HUVECs are dependent on ID‐1 down‐regulation and p53 up‐regulation.

### ID‐1 and P53‐mediated 2‐ME‐induced inhibitory effects on HIF‐1α expression in HUVECs

**Figure 5:**
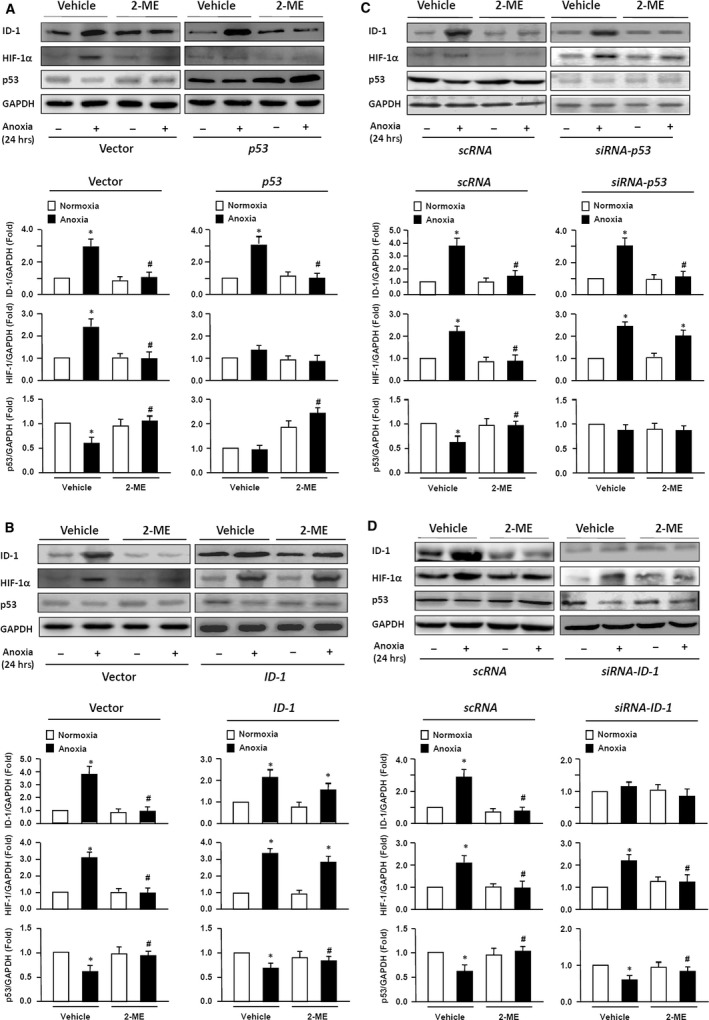
The roles of ID‐1 and p53 in the effects of 2‐ME on anoxia‐induced expression of HIF‐1α proteins in HUVECs. The HUVECs were transfected with p53 genes (**A**), siRNA of p53 (**B**), ID‐1 genes (**C**), siRNA of ID‐1 (**D**) or their empty vectors (Vector) or scramble sequence of RNA (scRNA) for 24 hrs. The cells were then added with 2‐ME or vehicle and subjected to normoxia (‐) or anoxia (+) incubation for 24 hrs. The protein expressions of ID‐1, HIF‐1α and p53 were analysed by Western blotting. GAPDH expression was used as the loading control. **A**,** B**,** C** and **D**, representative blots from six independent experiments are shown. Expressions of ID‐1, HIF‐1α and p53 proteins were quantified as folds of GAPDH expression in the cells subjected to normoxia with vehicle. Data are expressed as mean ± S.D. of six independent experiments (*n* = 6).*P <* *0.05 *versus* normoxia with Vehicle; ^#^ *P *<* *0.05 *versus* Anoxia with Vehicle.

Up to now, we have already demonstrated that 2‐ME could reduce HIF‐1α in VH rats as well as in anoxic HUVECs above. Then we wish to know whether 2‐ME reduces HIF‐1α mediated by ID‐1 and p53 in HUVECs. In shRNA‐ID‐1‐HUVECs, HIF‐1α level was not highly increased as scRNA‐HUVECs in response to 24 hrs anoxia culture. At the same time, pre‐treatment with 2‐ME showed a better intervention effect on HIF‐1α suppression. For ID‐1‐HUVECs, HIF‐1α level was raised much more than controls after anoxic culture. Moreover, 2‐ME treatment brought little effect on HIF‐1α suppression (Fig. 5A and B). Furthermore, we repeated the similar experiment on shRNA‐p53‐HUVECs. As we hypothesized, HIF‐1α was up‐regulated more, and 2‐ME pre‐treatment had less effect on HIF‐1α suppression than scRNA‐HUVECs, when cultured with anoxia. Overexpression of p53 in HUVECs down‐regulated the expression of HIF‐1α to a pretty lower level than that in the control, especially with anoxic culture (Fig. 5C and D). Interestingly, all of above manipulation of p53 and ID‐1 expression in HUVECs did not interfere with each other. Besides, p53 did not participate in 2‐ME‐mediated inhibition of anoxia‐induced phosphorylation of Akt1 (See Fig. S2). These results suggest that the inhibitory effects of 2‐ME on anoxia‐induced HIF‐1α expression in HUVECs are separately dependent on ID‐1 down‐regulation and p53 up‐regulation.

## Discussion

This study has revealed the importance of ID‐1 and p53 in endothelial cells in response to hypoxia‐induced angiogenesis. Either ID‐1 up‐regulation or p53 down‐regulation promotes HIF‐1α expression and angiogenesis, in the basal ganglia of intracranial VH rat model and HUVECs. The knowledge of ID‐1 protein is mainly from oncology researches. ID‐1 is a member of the ID protein family, proved to regulate cell differentiation, cell‐cycle progression and apoptosis 17. Kim *et al*. 10. found the possibility of cross talk between ID‐1 and HIF‐1α, either from normoxic or hypoxic conditions. ID‐1 can enhance HIF‐1α protein stability, nuclear translocation and transcriptional activity in breast cancer cells probably through ERK pathway. As a critical tumour suppressor protein, p53 has long been the study focus in the cancer researches. One involvement between p53 and HIF‐1α is the protein–protein interaction 18, existing as a molecular complex *in vivo* and *in vitro*. *in vivo*, p53 can repress the HIF‐1‐stimulated transcription on a sufficient level 19. This p53‐dependent suppression of HIF‐1 has been ascribed to a decrease in HIF‐1α stability 20, 21, 22.

ID‐1 and p53 are strongly implicated in regulation of angiogenesis. ID‐1 has been shown to induce angiogenesis through up‐regulation of HIF‐1α, and p53 has been reported as a suppressor of angiogenesis through inhibition of HIF‐1α. However, there are few reports showing the correlation between ID‐1 and p53 in angiogenesis. In one study on breast cancer cell, ID‐1 was reported to activate the Akt pathway by inhibition of phosphatase and tensin homologue deleted on chromosome 10 transcription through down‐regulation of p53 23, suggesting an ID‐1‐mediated change of p53 level. Although our *in vivo* experiments also showed the marginal down‐regulation of p53 after ID‐1 local overexpression, the *in vitro* study in HUVEC revealed that p53 level could not be changed by overexpression or knockdown of ID‐1. On the other hand, neither *in vivo* nor *in vitro* experiments showed any changes of ID‐1 levels after up‐ or down‐regulation of p53. Collectively, our present study indicates the simultaneous but independent role of p53 and ID‐1 in the regulation of HIF‐1α and angiogenesis.

The HIF‐1 is a transcription factor that interacts with hypoxia‐response elements. The transcription of over 60 genes can be up‐regulated by HIF‐1, many of them are in relevance with angiogenesis. One important insight into the mechanism of 2‐ME on anti‐angiogenesis is thought to be associated with HIF‐1α. It is speculated that 2‐ME block the transcription of HIF‐1a, as well as other proangiogenic genes dependent on HIF‐1a 24. There are reports suggest microtubule is the major target of 2‐ME that mediated HIF‐1a inhibition 25, 26. As a result, numerous angiogenic genes can be regulated by 2‐ME directly or indirectly. Previous studies have already demonstrated that 2‐ME can disrupt angiogenesis effectively at different stages in the formation of new blood vessels. Despite the studies in oncology, 2‐ME exhibited anti‐angiogenic activity in the corneal micropocket vascularization *in vivo* 12 and inhibited development of neovascularization in rat aortic ring assay 27, 28. From our data, 2‐ME plays an effective role in anti‐angiogenesis *in vivo* and *in vitro*. However, with ID‐1 overexpression or p53 knockdown, the effectiveness of 2‐ME was limited, restored MVDs and angiogenesis factors in the basal ganglia, as well as endothelial cells functions. In a breast cancer mouse model, Huh *et al*. confirmed that ID‐1 level was repressed by 2‐ME in tumour and vascular endothelial cells with a dose–effect relationship 29. Several reports showed that 2‐ME‐induced apoptosis in different cancer cell lines through p53 pathway up‐regulation 30, 31. In the present study, we first find that 2‐ME can suppress ID‐1 and increase p53 level in the VH brain tissue and hypoxic HUVECs, resulting in HIF‐1α suppression and low angiogenesis activation, indicating a novel medical drug for chronic ischaemia‐related intracranial vascular malformations. According to a Phase I clinical trial, the maximal 2‐ME administration dose is 1000 mg/day, and its half‐life is 10 hrs in the human body with a daily dose 32. In another trial, 2‐ME was given from 200‐1000 mg/day orally and last for 28 days in one circle and continued for a maximum of six cycles 33. Based on previous *in vivo* studies 9, 12, 34, 2‐ME injection at a dose of 60 mg/kg/day was safe and proper for the rats in this study.

Brain AVM is not so common, with young adults’ prevalence, but is associated with neurological morbidity and death 35. Treatment strategies for brain AVM include surgical resection, embolization and stereotactic radiotherapy. However, indication of these invasive treatments for un‐ruptured AVM is still under debate 36. Some types of brain AVM, such as those with large volume and deep drainage vein, are still associated with high risks, and the outcome of existing interventional treatments or radiotherapy is poor 36, 37, 38, 39. Recently, a report from the un‐ruptured brain arteriovenous malformations trial (ARUBA) indicated that medical management alone could be superior to interventional therapy, in purpose of preventing death or stroke in patients with un‐ruptured AVM 36. This trial highlights alternative medical treatments to control AVM development without invasive therapy, leading future investigations of medicine for such diseases. In the current study, we use the VH rat model to simulate the conditions of human brain AVMs. This model was firstly introduced by Lawton *et al*. in 1997 6, highlighting the VH as the cause of aberrant angiogenesis. Because of the surgical difficulties of common carotid artery‐external jugular vein (CCA‐EJV) anastomosis, this kind of model was mainly performed on rats. On the other hand, the use of HUVECs *in vitro* can well represent the human species, confirming the mechanism of 2‐ME as the complementarity of *in vivo* data. There are several limitations in this study. Firstly, we used a local *in vivo* injection of lentiviral vectors to knockdown p53 or overexpress ID‐1 genes, but did not use the p53 knockout and ID‐1 transgenic animals. Secondly, as it is very difficult to obtain the endothelial cells from rat intracranial vessels, we used culture HUVEC to perform *in vitro* experiments. There might be some differences between the two cells types. Thirdly, we focused here the roles of ID‐1 and p53 in the effects of 2‐ME on HIF‐1 and angiogenesis. Other angiogenesis‐regulating molecules such as Akt and Notch may be also involved and should be investigated in our future study.

**Figure 6:**
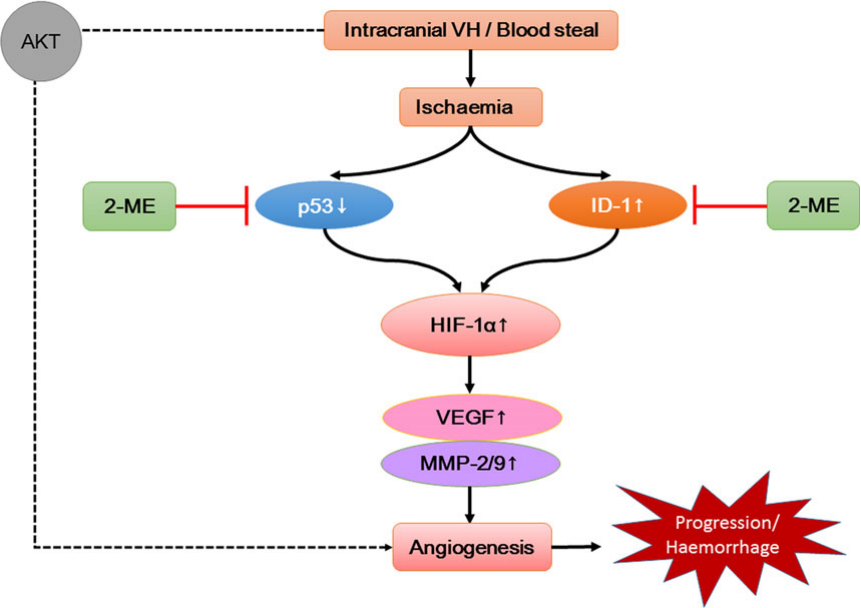
Proposed mechanism underlying the inhibitory effects of 2‐ME on anoxia‐induced angiogenesis.

We present here novel *in vivo* findings that 2‐ME treatment reduces the progression of brain tissue angiogenesis in the intracranial VH rats mediated by ID‐1 and p53 regulations. *in vitro* studies further confirm the role of ID‐1 and p53 in 2‐ME effectiveness. The proposed mechanism was displayed in Figure 6D. Taken together, these observations indicate that 2‐ME treatment may be a potential alternative medical treatment for un‐ruptured AVM patients, which still need future clinical investigations.

## Author contributions

L.C, Y.M. and W.Z. conceived and designed experiments. X.Z. and Z.W. performed *in vivo* experiments. L.Z., J.Y. and C.Y. performed *in vitro* experiments. X.Z., L.Z. and J.Y. involved in data analysis. J.S. involved in clinical data. X.Z. and L.Z. wrote and edited the manuscript. L.C. involved in approval of the submitted version.

## Conflicts of interest

The Authors declare that there is no conflict of interest.

## Supporting information


**Figure S1** Operation schematic diagram for rat VH model and grouping illustration of the experiment.

**Figure S2** The roles of p53 in 2‐ME‐mediated inhibition of anoxia‐induced phosphorylation of Akt1.The HUVECs were transfected with p53 genes or siRNA of p53 for 24 hrs.
