# Correction to “Endogenous Hormone 2‐Methoxyestradiol Suppresses Venous Hypertension‐Induced Angiogenesis Through Up‐ and Down‐Regulating p53 and ID‐1”

**DOI:** 10.1111/jcmm.71100

**Published:** 2026-03-30

X. Zou, L. Zhang, J. Yuan, et al., “Endogenous Hormone 2‐Methoxyestradiol Suppresses Venous Hypertension‐Induced Angiogenesis Through Up‐and Down‐Regulating p53 and ID‐1,” *Journal of Cellular and Molecular Medicine* 22(2018): 957–967. https://doi.org/10.1111/jcmm.13399

In Xiang Zou et al. one immunohistochemistry panel of Figure 3B was misplaced in Figure 1A due to a technical error during image preparation. The correct Figure 1 is shown below. The authors confirm all results and conclusions of this article remain unchanged.

**FIGURE 1:**
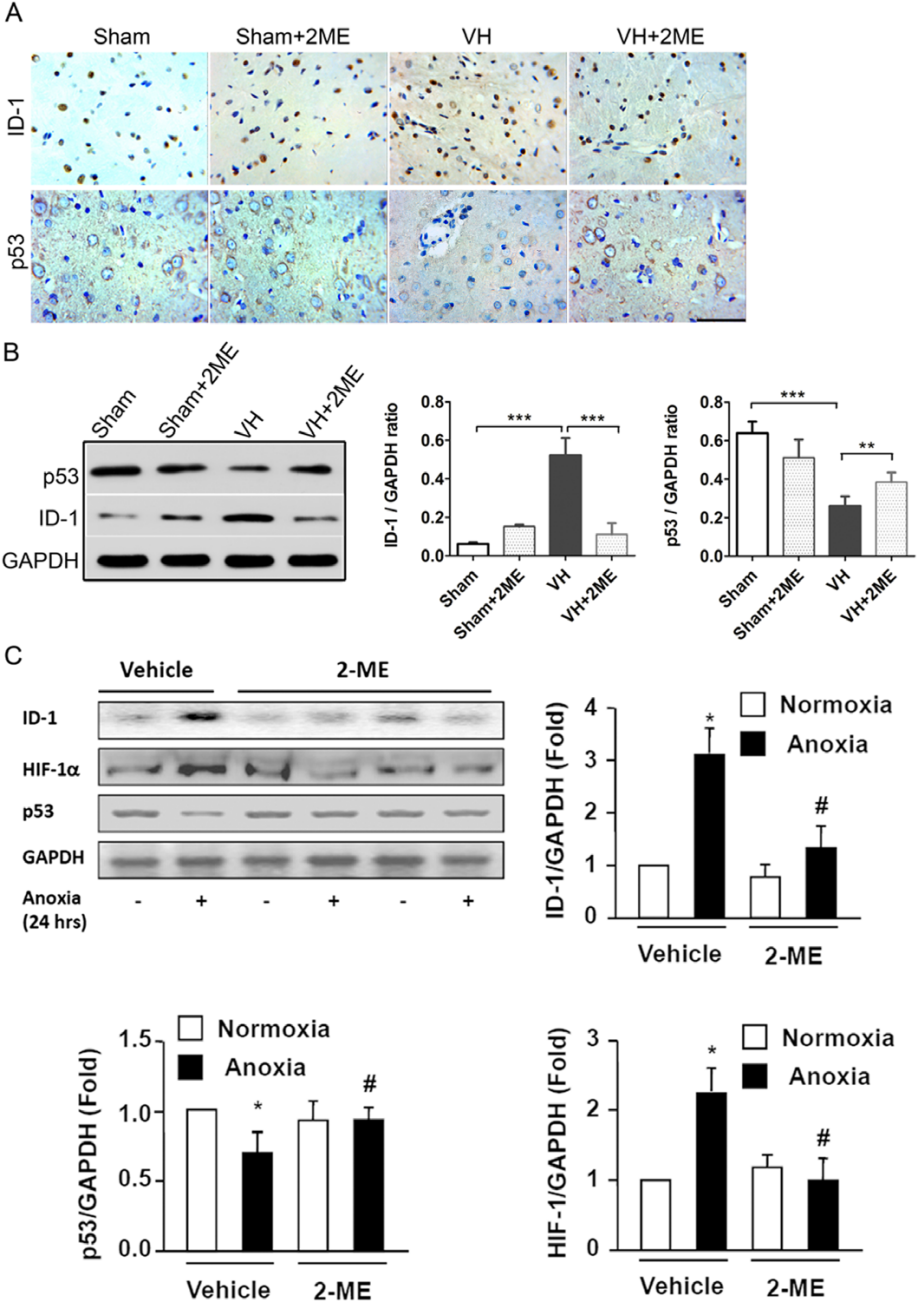
The effects of 2‐ME treatment on the expression ID‐1 and p53 proteins in the basal ganglia after intracranial VH modelling.(A) Immunohistochemistry staining of ID‐1 and p53 proteins in basal ganglia of the Sham and intracranial VH rats, with or without 2‐ME treatment. Scale bar: 50 μm. (B) Western blot analysis of ID‐1 and p53 proteins in the rats of Sham, Sham+2ME, VH and VH+2ME groups. **p < 0.01, ***p < 0.001; *n* = 6 per group; mean ± S.D. (C) Expressions of ID‐1, p53 and HIF‐1α proteins were quantified as folds of GAPDH expression in the cells subjected to normoxia with vehicle. Data are expressed as mean ± S.D. of six independent experiments (*n* = 6). *p < 0.05 versus normoxia with Vehicle; # *p* < 0.05 versus Anoxia with Vehicle.

