# Supplementary material for: Endogenous hormone 2‐methoxyestradiol suppresses venous hypertension‐induced angiogenesis through up‐ and down‐regulating p53 and id‐1
Source: J Cell Mol Med. 2017 Nov 29;22(2):957–67. doi: 10.1111/jcmm.13399 (PMC5783857; doi:10.1111/jcmm.13399)
Supplement: Supplementary file 1 — Figure S1 Operation schematic diagram for rat VH model and grouping illustration of the experiment. [file JCMM-22-957-s001.pdf]

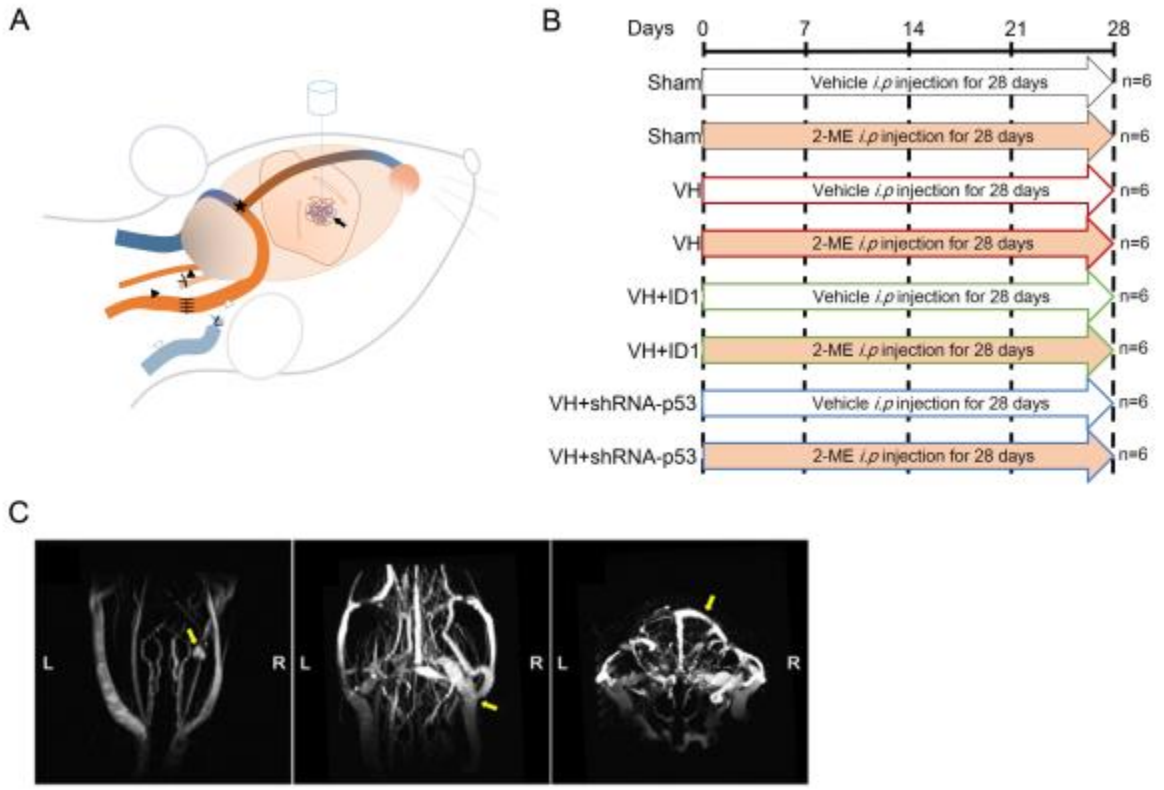

Figure_1_SuppInfo. Operation schematic diagram for rat VH model and grouping illustration of the experiment. (**A**) Operation schematic diagram for rat VH model. Black triangle: right internal carotid artery. White triangle: right external jugular vein. Black star: torcular. Black arrow: right basal ganglia. (**B**) Animal grouping based on modeling, ID-1 overexpression or p53 knockdown, and 2-ME intervention. (**C**) Magnetic resonance angiography of VH model. Left: Horizontal view of neck magnetic resonance angiography showing the anastomotic stoma (yellow arrow) of proximal common carotid artery and the distal external jugular vein 2 days after surgery. Middle: horizontal view of head magnetic resonance angiography showing dilation of right distal external jugular vein (yellow arrow) in a horizontal position 14 days after surgery. Right: coronal view of head magnetic resonance angiography showing dilation of right transverse sinus (yellow arrow) in a horizontal position 14 days after surgery.

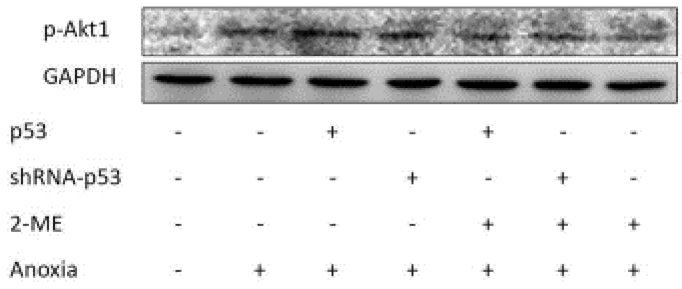

SI Figure 2. The roles of p53 in 2-ME-mediated inhibition of anoxia-induced phosphorylation of Akt1.The HUVECs were transfected with p53 genes or siRNA of p53 for 24 hours. The cells were then added with or without 2-ME and subjected to normoxia (-) or anoxia (+) incubation for 24 hours. The phosphorylation of Akt1 was analyzed by Western blotting. GAPDH expression was used as the loading control. Representative blots from three independent experiments are shown.
