# Supplementary material for: Endogenous hormone 2‐methoxyestradiol suppresses venous hypertension‐induced angiogenesis through up‐ and down‐regulating p53 and id‐1
Source: J Cell Mol Med. 2017 Nov 29;22(2):957–67. doi: 10.1111/jcmm.13399 (PMC5783857; doi:10.1111/jcmm.13399)
Supplement: Supplementary file 2 — Figure S2 The roles of p53 in 2‐ME‐mediated inhibition of anoxia‐induced phosphorylation of Akt1.The HUVECs were transfected with p53 genes or siRNA of p53 for 24 hrs. [file JCMM-22-957-s002.pdf]

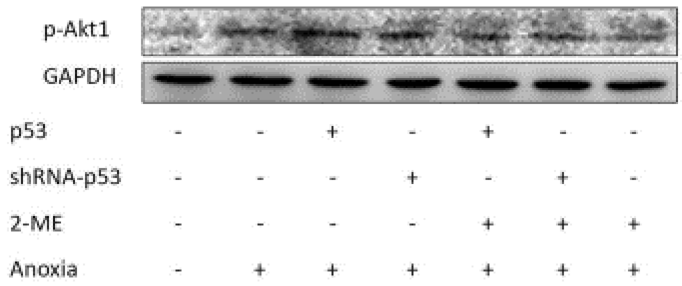

Figure_2_SuppInfo. The roles of p53 in 2-ME-mediated inhibition of anoxia-induced phosphorylation of Akt1.The HUVECs were transfected with p53 genes or siRNA of p53 for 24 hours. The cells were then added with or without 2-ME and subjected to normoxia (-) or anoxia (+) incubation for 24 hours. The phosphorylation of Akt1 was analyzed by Western blotting. GAPDH expression was used as the loading control. Representative blots from three independent experiments are shown.
